# Accuracy of Component Orientation and Leg Length Adjustment in Total Hip Arthroplasty Using Image-free Navigation

**DOI:** 10.2174/1874325001711011432

**Published:** 2017-12-28

**Authors:** Yu Takeda, Shigeo Fukunishi, Shoji Nishio, Yuki Fujihara, Shinichi Yoshiya

**Affiliations:** Hyogo College of Medicine, Department of Orthopedic Surgery 1-1, Mukogawa-cho, Nishinomiya, Hyogo, Japan

**Keywords:** Component orientation, CT evaluations, Leg length, Total Hip Arthroplasty, Image-free, Navigation

## Abstract

**Purpose:**

The purpose of this study was to examine the accuracy of implant orientation and leg length in total hip arthroplasty (THA) with an image-free navigation system based on a comparison of the intraoperative navigation and postoperative CT evaluations.

**Material and Methods:**

A consecutive series of 111 patients (118 hips) who underwent THA using the current version of the image-free navigation system constituted the basic study population. Subsequently, a total of 101 patients (108 hips) meeting the inclusion and exclusion criteria were selected as study subjects for the analysis. THA was performed using an image-free navigation system that was capable of adjusting both the prosthetic position and leg length. Postoperative CT examination was performed for all study subjects, and the prosthetic position and leg length were measured on CT images using the image analysis software. Subsequently, the intraoperative navigation results and the corresponding values obtained from the postoperative CT measurements were compared to test the accuracy of the navigation system.

**Results:**

The average discrepancies between the intra- and postoperative assessments were 6.8°, 3.7°, and 5.7° for cup anteversion, cup inclination, and stem anteversion, respectively. The corresponding value in leg length averaged 4.1 mm.

**Conclusion:**

Average discrepancies between the intra- and postoperative measurements were less than 10° in all prosthetic alignment parameters and less than 5 mm in leg length. Intraoperative assessments with the use of the image-free navigation in THA could afford satisfactory result.

## 1 INTRODUCTION

Previous studies have indicated that implant positioning is an important factor influencing the postoperative outcome in Total Hip Arthroplasty (THA). Malposition of the implant may induce an increased risk for postoperative complications such as dislocation, prosthetic impingement, restricted range of motion, polyethylene wear, early aseptic loosening and leg length discrepancy [1, 2]. Among the measures to improve the surgical accuracy, the use of a computer-assisted navigation system has been shown to help achieve optimal implant alignment [1, 3-5]. The THA navigation systems that are currently in use can be divided into two categories based on operational principle: computed tomography based and image-free [1, 6, 7]. The image-free navigation system, however, has been known to raise concerns over its accuracy and reproducibility because the three-dimensional geometry of the pelvis (anterior pelvic plane; APP) is determined by three anatomical landmarks (bilateral anterior superior iliac spine (ASIS) and pubic tubercle) located by palpation. We have been using an image-free navigation system in THA since 2006. Based on a review of our clinical experiences using previous versions of image-free navigation systems (Orthopilot ver1.1 and Orthopilot THAplus B/BRAUN-Aesculap, Germany), the co-author of the present study has reported satisfactory accuracy and consistency in the assessment of cup positioning [5, 6]. However, the previous version of the navigation system was not equipped with a tool to evaluate stem anteversion and leg length. Since December 2011, the authors have used a revised version (Orthopilot THAPro), which is capable of navigating stem anteversion alignment and leg length including cup orientation. Achieving an improvement in overall alignment can be expected; however, there have been studies examining and reporting the accuracy of intraoperative navigation, an accuracy assessment for this revised version has not been reported in previous literature. Additionally, several studies have reported on the accuracy of implant positioning using Computed Tomography (CT) examination; however, there have only been a few reports on measurement with a three-dimensional (3D) template system. The purpose of this study was to assess the accuracy of the current version of the image-free navigation system in detail using a 3D template system by comparing measurement values obtained from intraoperative navigation to a postoperative CT evaluation with a 3D template system. It was hypothesized that reasonable accuracy would be attained with the use of this system.

## 2 MATERIALS AND METHODS

111 Japanese patients were included in this retrospective and consecutive study. This study was approved by our institutional review board and informed consent about the surgery procedure and the use of CT for evaluation of implant positioning pre- and post-surgery was obtained from all patients.

### 2.1 Study Population

111 patients (118 hips) underwent primary THA using an image-free navigation system (Orthopilot THApro) during the period from December 2011 to November 2016. A postoperative CT examination was performed for all patients to assess the implant positioning. The exclusion criteria were previous total knee arthroplasty and presence of substantial pelvic deformity associated with previous pelvic osteotomy, revision hip surgery and infection. In total, 10 out of the 118 hips were excluded from the study due to the exclusion criteria. Consequently, 108 hips in 101 patients met the inclusion/exclusion criteria and were subjected to the analysis of this study. There were 19 male and 82 female patients with a mean age of 65.2 years (range: 52 to 86 years) and a mean body mass index (BMI) of 23.2 ± 3.15 kg/m^2^ (range: 14.4 to 32.7 kg/m^2^).

Patients with a BMI of $\underline{\underline{>}}$ 25 were classified as obese (25 patients, 25 hips) and those with a BMI < 25 as non-obese (76 patients, 83 hips) in accordance with the obesity criteria for Japan [8, 9]. Hip pathologies in this study population included osteoarthritis due to hip dysplasia in 92 cases (96 hips) cases and osteonecrosis in 9 cases (12 hips) (Table **1**).

### 2.2 Surgical Procedure

Surgery was performed by two senior surgeons (SF and YT) who are experienced with using the image-free navigation system. Before surgery, the screw with the tracker device was placed into the ipsilateral ilium with the patient in supine position. In addition, another tracker device (femoral clamp) was attached to the greater trochanter of the femur during surgery. The surgical approach was either the modified-Hardinge approach with the patient positioned in the lateral position or the anterior-lateral approach in the supine position. The modified-Hardinge approach was employed for 71cases (76 hips) and the anterior-lateral approach was employed for 30 cases (32 hips). Regarding the leg length, the length of the operated leg was adjusted to the value obtained from the radiograph data of the contralateral leg. The implanted prosthetic system was composed of a cementless cup (Plasma cup BTM, B/Braun-Aesculap, Germany), a cementless stem (BicontactTM, B/Braun-Aesculap, Germany), a ceramic 32-mm head, and a ceramic liner from the same manufacturer.

### 2.3 Intraoperative Navigation

Image-free navigation relies on an anterior pelvic plane (APP) defined by the three obligatory bony landmarks (bilateral ASISs and pubic tubercle). These landmarks are palpated by a navigation pointer during the registration process. During the procedure, cup anteversion (AV) and inclination angles were calculated in reference to the APP. Regarding the femoral side, the intraoperative stem anteversion (AT) angles were calculated using the functional femoral plane (FFP) as a reference. The FFP plane was defined by the mid-point of the ankle malleoli, the mid-point of the knee, and the hip center. Regarding the leg length discrepancy (LLD), the intraoperative LLD were calculated based on the distance from the center of the hip to the femoral clamp. After insertion of the trial implant, the leg length change was presented on the navigational screen, and the surgeon could intraoperatively adjust the leg length.

### 2.4 Postoperative Evaluation

**Fig. (1):**
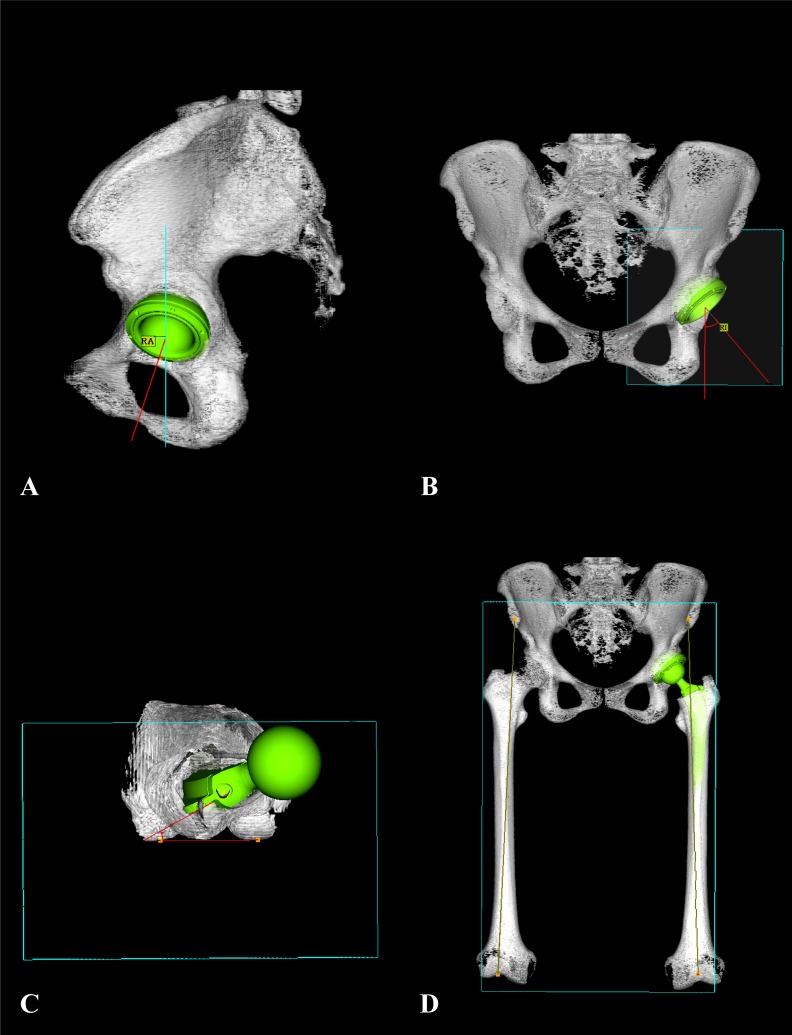
Postoperative cup and stem alignment as well as change in leg length were assessed using CT with 3D-Template system. A: Cup anteversion, B: Cup inclination, C: Stem anteversion, D: Leg length.

For assessment of the postoperative implant orientation and leg length, all included patients underwent pre- and postoperative CT examinations. A helical CT scan (Somatom; Siemens, Munich, Germany) providing images with a 3-mm slice interval from ASIS to the knee was performed for all cases. Postoperative cup and stem position were assessed using a 3D-Template system (ZedHip, LEXI, Japan) after CT examination. In this measurement, cup anteversion and inclination were evaluated in reference to APP, and stem anteversion was evaluated using the condylar axis as a reference line (Fig. **1**). During the calculation of the angles for prosthetic alignment, anatomical angles obtained from the CT measurements were converted to an angle for radiological definition as used in the navigation assessment to enable fair comparison. Leg length was measured on the functional pelvic plane after repositioning using the 3D-Template system, and it was then assessed by the distance from the ASIS to the knee center. Postoperative leg length change was defined as the difference between pre- and postoperative CT measurement values (Fig. **1**). The 3D-Template system was used to match the pre- and postoperative CT digital image. In order to test the accuracy of the navigation system for leg length, the intraoperative navigation results and the corresponding values obtained from the postoperative CT measurements were compared.

### 2.5 Statistical Analysis

All statistical analyses were conducted using SPSS (version 19; IBM SPSS Statistics, Inc, Chicago, IL) for Windows. Continuous data were analyzed using the nonparametric Mann-Whitney U test. *P*<0.01 was considered significant. In order to examine the accuracy of the navigation assessment, the discrepancy (absolute difference) between the intraoperative navigation assessment and the postoperative CT evaluation was calculated for implant orientation parameters (cup anteversion/inclination and stem anteversion) and leg length. In addition, the correlation between the two measurements was statistically analyzed using the Pearson correlation coefficient test.

## 3 RESULTS

### 3.1 Complications

There were no major complications, such as dislocation, deep infection, and revision THA, encountered during the study period. The average follow-up period was 2 years 5 months (range: 6 months to 6 years).

In addition, there were no complications related to the navigation procedures such as superficial infection or nerve damage at the site of tracker insertion.

### 3.2 Statistical Analysis in Subgroup

#### 3.2.1 BMI

There were no significant differences between the obese and non-obese groups for the absolute discrepancy regarding cup anteversion (AT) and inclination (CI), stem anteversion (AT), or leg length (LLD) (Table **2**).

#### 3.2.2 Surgical Position

Similarly, there were no significant differences between the lateral position and supine position groups for the absolute discrepancy regarding cup anteversion and inclination, stem anteversion, or leg length (Table **3**).

#### 3.2.3 Implant Orientation

**Fig. (2):**
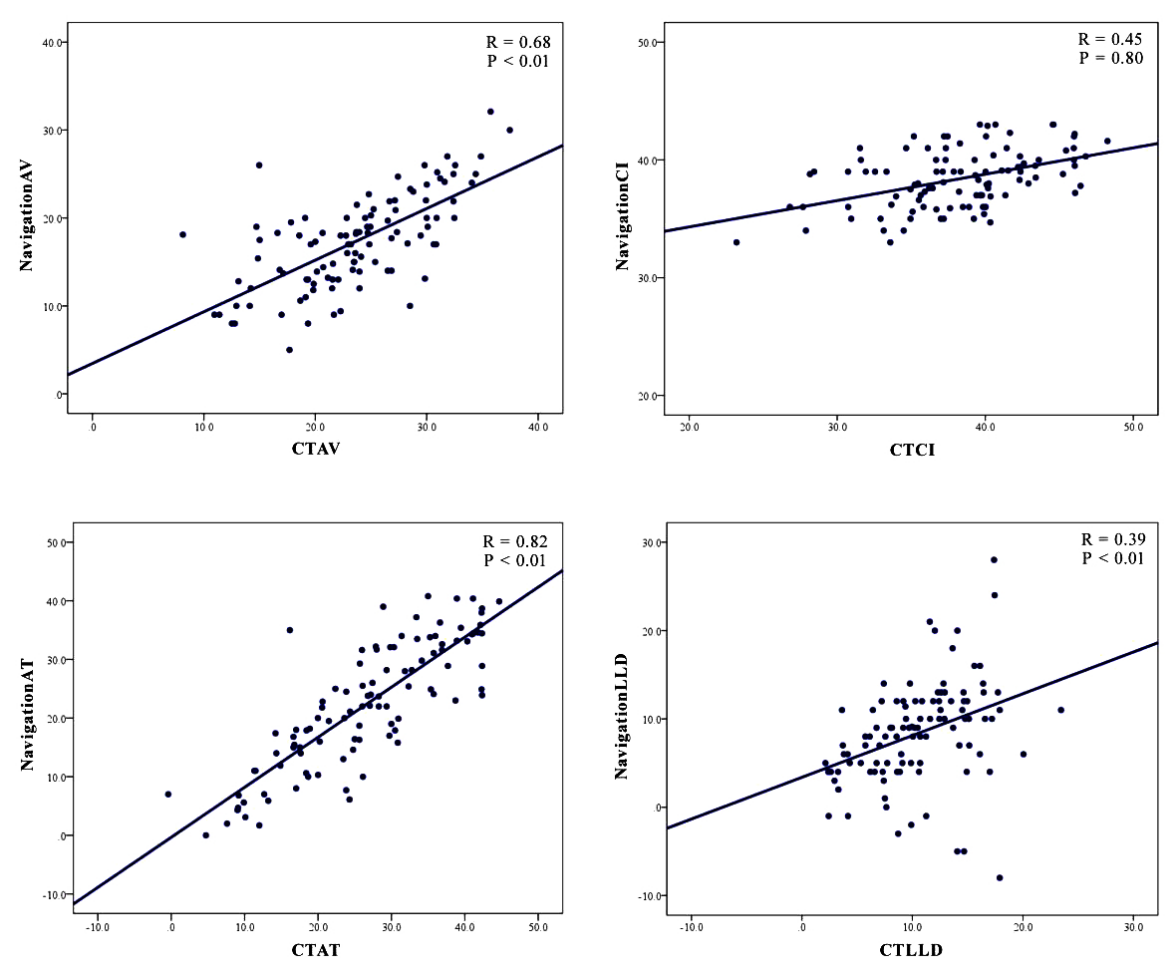
Results of the Pearson correlation coefficient between the intraoperative values and CT measurement values. (Cup anteversion: AV, Cup inclination: CI, Stem anteversion: AT, Leg length discrepancy: LLD).

Intraoperative assessment by the navigation system indicated that the cup anteversion (AV) and cup inclination (CI) values, stem anteversion (AT) averaged 17.3° ± 5.3° (range: 5° to 32.1°) and 38.3° ± 2.4° (range: 33.0° to 43.0°), 22.5° ± 10.6° (range: 0° to 40.8°). Postoperative CT evaluation indicated that the cup anteversion and cup inclination, stem anteversion values averaged 23.4° ± 6.2° (range: 8.12° to 37.4°) and 38.4° ± 5.0° (range: 23.2° to 48.2°), 26.5° ± 10.3° (range: -0.41° to 44.7°). The average absolute discrepancies between the intraoperative and CT measurement were 6.9° ± 3.6 (range: 0.3° to 18.1°) for cup anteversion, 3.6° ± 2.7 (range: 0.05° to 10.6°) for cup inclination, and 5.8° ± 4.6 (range: 0.01° to 18.8°) for stem anteversion (Table **4**). The Pearson correlation coefficients were 0.68 for cup anteversion and 0.45 for cup inclination, 0.82 for stem anteversion (Fig. **2**).

#### 3.2.4 Leg Length

Intraoperative elongation of leg length (LLD) as assessed by the navigation system averaged 8.2 mm ± 5.7 mm (range: -8 mm to 28 mm), while the corresponding value measuring the pre- and postoperative CT images was 10.2 mm ± 4.5 mm (range: 2.1 mm to 23.4 mm). When the intraoperative and CT evaluation results were compared, the average absolute discrepancy between the two measurements was 4.3 ± 4.5 mm (range: 0.02 mm to 14.0 mm) (Table **4**). The Pearson correlation coefficient was 0.39 (Fig. **2**).

## 4 DISCUSSION

In the present study, all parameters, including cup alignment, stem alignment, and leg length in THA performed with the image-free navigation system, were subjected to accuracy analysis. With regard to cup positioning, the average absolute discrepancies between the intraoperative and CT measurement results were 6.9°±3.6 for anteversion and 3.6°±2.7 for inclination. Regarding the factors affecting the accuracy of cup alignment, this system could ensure accurate and reproducible acetabular cup positioning. However, the accuracy was less than what was reported in a previous report which used a CT navigation system [6, 7]. One of the potential sources of errors in the use of this system is percutaneous registration of bony landmarks. Several papers have analyzed the effect of subcutaneous tissue thickness on the intraoperative assessment error using BMI and soft tissue thickness values as influential factors [2, 8, 10, 11]. In those studies, the presence of soft tissue between the skin and the bony landmarks has been pointed out as a major source of registration error. However, in the present study, there was no significant difference between the obese and non-obese groups. Furthermore, the correlation between the intraoperative navigation value and CT value was low in cup inclination. This result suggested that accurate percutaneous registration of bony landmarks was limited even in non-obese patients. Additionally, the error of initial registration procedure has a large influence on the intraoperative position of the cup, thus it was one of the most obvious technical limitations of the imageless navigation system based on bony landmark [10]. In the assessment of stem alignment, only a few studies have examined the accuracy of femoral stem orientation during navigated THA [12-14]. In the present study, the average absolute discrepancy between the intraoperative and CT measurement values was 5.7°±4.5with a Pearson correlation coefficient of 0.82, showing that a high accuracy was achieved by the navigation system used in this study. In the comparison of intraoperative navigation and postoperative CT results, the difference in reference plane between the two measurements should be taken into consideration. Stem anteversion angle is calculated in reference to the FFP in the intraoperative navigation measurement, while postoperative stem anteversion with CT analysis adopts the femoral condylar axis as a reference. As for the effect of this difference in the reference on anteversion measurement, Turley et al. assessed the validity of the measurement principle employed in the navigation system and concluded that the navigation results can be reasonably compared to the values measured on CT images [15]. There have been several studies examining leg length change after THA with image-free navigation [16-18, 19]. Previous study examined the accuracy of leg length adjustment in THA using a previous version of image-free navigation in which the leg length was intraoperatively measured without a femoral clamp attached to the femur. Relatively accurate leg length adjustment was feasible with the previous study [17]; however, in most of the previous studies, including our previous study, leg length measurement was evaluated on anterior-posterior radiographs [15-17, 20]. The measurement based on pelvic radiograph can be associated with potential sources of errors arising from several factors such as rotation of the pelvis and femur, as well as contracture of the hip and knee joint [19]. In the present study, leg length was measured as the distance from ASIS to knee center using the functional pelvic plane after reposition in the 3D-Template system. With this measurement method, it was possible to eliminate some of the measurement errors, and it provides improved measurement accuracy. Consequently, the difference in leg length discrepancy between intraoperative navigation measurement and CT evaluation averaged 4.1 mm ± 4.4. The potential source of assessment errors in the intraoperative assessment, it has been demonstrated that loosening of the device (femoral clamp attached to the greater trochanter) can be a factor inducing error. In addition, there are concerns that cases with the differences may occur because of the different measurement methods between the intra- and post-operative evaluation.

There are limitations of the study as follows: First, inter- and intra-examiner reliabilities in intra- and postoperative measurements were not quantitatively assessed. Second, the CT evaluation may not have provided absolutely accurate value while the accuracy assessment was based on the discrepancy between the navigation and CT evaluations.

Third, there was no control date, therefore it was not possible to evaluate and compare with the conventional technique for accuracy of implant orientation in this study. Finally, it was not possible to define the factors that caused large errors. Overall, the present study showed that the use of an image-free navigation system in THA could attain satisfactory accuracy for intraoperative adjustment of cup positioning/alignment, stem alignment, and leg length. A significant clinical result was achieved for all patients, and although this system has some inherent sources of inaccuracy and inconsistency, the obtained results have shown that an image-free navigation system can be a valuable tool to improve the accuracy of the THA procedure.

## CONCLUSION

Accuracy of intraoperative assessment of implant positioning and leg length was evaluated by comparing the navigation results to the values derived from postoperative CT examination. The average discrepancies between the intra- and postoperative measurements were less than 10° in all prosthetic alignment parameters and less than 5 mm in leg length. From moderate to high correlation was demonstrated between the intra- and postoperative measurement values. Intraoperative assessments with the use of the image-free navigation in THA could afford satisfactory clinical results.

## Figures and Tables

**Table 1:** Demographic data.

Parameter	N	Mean	Range
Gender (male/female)	19/82		
Age (years)		65.2	52 - 86
BMI (kg/m2) obese/nonobese	25/76	23.2 ± 3.15	14.4 - 32.7
Surgical position Lateral/supine (number of hips)	76/32		
Diagnosis OA/ ON (number of hips)	96 /12		

**Table 2:** Comparison of the implant position in the obese and nonobese groups.

Parameter (Absolute Discrepancy)	Obese Group (*n*=25)	Nonobese Group (*n*=76)	* P*
Cup anteversion	6.8° ± 3.4	6.8° ± 3.6	0.99
Cup inclination	3.8° ± 2.7	3.7° ±2.7	0.85
Stem anteversion	6.2° ± 4.8	5.5° ±4.4	0.59
Leg length	3.2 mm ±2.8	4.4 mm ±4.7	0.47

**Table 3:** Comparison of the implant position in the lateral position and supine position groups.

Parameter (Absolute Discrepancy)	Lateral Group (*n*=71)	Supine Group (*n*=30)	* P*
Cup anteversion	6.7° ± 3.6	7.1° ± 3.5	0.61
Cup inclination	3.6° ±2.6	3.8° ± 2.7	0.67
Stem anteversion	6.1° ±4.6	4.7° ± 4.0	0.13
Leg length	4.4 mm ±4.7	3.6 mm ±3.6	0.39

**Table 4:** Results of intraoperative navigation values and postoperative CT measurement values.

	Navigation Values	CT Values	Absolute Discrepancy	Correlation Coefficient
Cup anteversion	17.3° ± 5.3	23.46° ± 6.2	6.8° ± 3.6	0.68
Cup inclination	38.3°± 2.4	38.4° ± 5.0	3.7° ± 2.7	0.45
Stem anteversion	22.5°± 10.6	26.5° ± 10.3	5.7° ± 4.5	0.82
Leg length discrepancy	8.2mm ± 5.7	10.2mm ± 4.5	4.1mm ± 4.4	0.36

## References

[1] Kalteis T., Handel M., Bäthis H., Perlick L., Tingart M., Grifka J. (2006). Imageless navigation for insertion of the acetabular component in total hip arthroplasty: Is it as accurate as CT-based navigation?. J. Bone Joint Surg. Br..

[2] Hohmann E., Bryant A., Tetsworth K. (2011). A comparison between imageless navigated and manual freehand technique acetabular cup placement in total hip arthroplasty.. J. Arthroplasty.

[3] Liu Z., Gao Y., Cai L. (2015). Imageless navigation *versus* traditional method in total hip arthroplasty: A meta-analysis.. Int. J. Surg..

[4] Lass R., Kubista B., Olischar B., Frantal S., Windhager R., Giurea A. (2014). Total hip arthroplasty using imageless computer-assisted hip navigation: A prospective randomized study.. J. Arthroplasty.

[5] Sendtner E., Schuster T., Wörner M., Kalteis T., Grifka J., Renkawitz T. (2011). Accuracy of acetabular cup placement in computer-assisted, minimally-invasive THR in a lateral decubitus position.. Int. Orthop..

[6] Sugano N., Takao M., Sakai T., Nishii T., Miki H. (2012). Does CT-based navigation improve the long-term survival in ceramic-on-ceramic THA?. Clin. Orthop. Relat. Res..

[7] Iwana D., Nakamura N., Miki H., Kitada M., Hananouchi T., Sugano N. (2013). Accuracy of angle and position of the cup using computed tomography-based navigation systems in total hip arthroplasty.. Comput. Aided Surg..

[8] Tsukada S., Wakui M. (2010). Decreased accuracy of acetabular cup placement for imageless navigation in obese patients.. J. Orthop. Sci..

[9] Matsuzawa Y., Nakamura T., Takahashi M. (2002). New Criteria for ‘Obesity Disease’.. Jpn. Circ. J..

[10] Parratte S., Argenson J.N. (2007). Validation and usefulness of a computer-assisted cup-positioning system in total hip arthroplasty. A prospective, randomized, controlled study.. J. Bone Joint Surg. Am..

[11] Hohmann E., Bryant A., Tetsworth K. (2012). Anterior pelvic soft tissue thickness influences acetabular cup positioning with imageless navigation.. J. Arthroplasty.

[12] Dorr L.D., Malik A., Dastane M., Wan Z. (2009). Combined anteversion technique for total hip arthroplasty.. Clin. Orthop. Relat. Res..

[13] Hayashi S., Nishiyama T., Fujishiro T., Hashimoto S., Kanzaki N., Nishida K., Kuroda R., Kurosaka M. (2013). Evaluation of the accuracy of femoral component orientation by the CT-based fluoro-matched navigation system.. Int. Orthop..

[14] Renkawitz T., Sendtner E., Grifka J., Kalteis T. (2008). Accuracy of imageless stem navigation during simulated total hip arthroplasty.. Acta Orthop..

[15] Turley G.A., Ahmed S.M., Williams M.A., Griffin D.R. (2012). Validation of the femoral anteversion measurement method used in imageless navigation.. Comput. Aided Surg..

[16] Weber M., Woerner M., Springorum R., Sendtner E., Hapfelmeier A., Grifka J., Renkawitz T. (2014). Fluoroscopy and imageless navigation enable an equivalent reconstruction of leg length and global and femoral offset in THA.. Clin. Orthop. Relat. Res..

[17] Nishio S., Fukunishi S., Fukui T., Fujihara Y., Yoshiya S. (2011). Adjustment of leg length using imageless navigation THA software without a femoral tracker.. J. Orthop. Sci..

[18] Renkawitz T., Schuster T., Grifka J., Kalteis T., Sendtner E. (2010). Leg length and offset measures with a pinless femoral reference array during THA.. Clin. Orthop. Relat. Res..

[19] Kjellberg M., Al-Amiry B., Englund E., Sjödén G.O., Sayed-Noor A.S. (2012). Measurement of leg length discrepancy after total hip arthroplasty. The reliability of a plain radiographic method compared to CT-scanogram.. Skeletal Radiol..

[20] Ellapparadja P., Mahajan V., Deakin A.H., Deep K. (2015). Reproduction of hip offset and leg length in navigated total hip arthroplasty: How accurate are we?. J. Arthroplasty.

